# Protective nutrition strategy in the acute phase of critical illness: why, what and how to protect

**DOI:** 10.3389/fnut.2025.1555311

**Published:** 2025-05-09

**Authors:** Youquan Wang, Yanhua Li, Nan Li, Yuting Li, Hongxiang Li, Dong Zhang

**Affiliations:** Department of Critical Care Medicine, The First Hospital of Jilin University, Changchun, China

**Keywords:** critical illness, intensive care, enteral nutrition, feeding intolerance, feeding strategy, refeeding syndrome, acute mesenteric ischemia

## Abstract

Nutritional support is crucial for critically ill patients. Recent clinical studies suggest that both overfeeding during the acute phase of critical illness and overly conservative or delayed nutritional therapy can pose significant risks. Given substantial individual variability among critically ill patients, it is challenging to prescribe universally applicable and objective feeding strategies; Instead, we pointed out which nutritional interventions were harmful. We also summarized the reasons for protective nutrition, and elaborated the advantages of protective nutrition from three perspectives: gastrointestinal protection, nutritional protection and metabolic protection. In particular, it is emphasized that overfeeding will lead to metabolic disorders, such as mitochondrial dysfunction, autophagy inhibition, ketogenic inhibition, hyperglycemia, insulin resistance, etc. These detrimental processes can exacerbate one another, contributing to multiple organ dysfunction syndrome and poorer clinical outcomes. We also propose protective nutrition strategies comparable to lung protective ventilation strategies, which may benefit patients. Vigilant monitoring during nutritional implementation is also paramount, enhancing awareness of adverse events for early diagnosis and intervention to mitigate their harm.

## 1 Introduction

During the acute phase of critical illness, there is an increase in catabolism characterized by intense inflammatory response, glycogen and protein breakdown, and dysregulation of metabolic control (1). These multifaceted factors may contribute to malnutrition and the development of intensive care unit (ICU)-acquired weakness, which in turn can prolong mechanical ventilation and hospitalization, and even increase ICU and in-hospital mortality (2, 3). The uncontrollable hypercatabolic state during the acute phase of critical illness prompted recognition of the importance of nutrition, aiming to counteract the catabolic metabolism and thus improve the nutritional status and clinical outcomes of critically ill patients (4, 5). Previous studies have also showed that early and full nutritional therapy associated with reduced infectious complications and ICU-related complications in critically ill patients, and can even reduce 60-day mortality (6–8). However, these studies are all observational in nature, making it difficult to avoid bias. The better prognosis observed may not be attributable to full nutritional therapy, but rather to the fact that these patients had milder conditions and better feeding tolerance. Therefore, imposing a causal relationship in this context is highly misleading.

Given the uncertainty of the relationship between nutritional therapy and prognosis, several randomized controlled trials (RCTs) have been conducted in recent years to explore the true relationship between the two, and these studies have shown that early full nutrition is not beneficial and may even be harmful (9–15). This finding, contrary to previous thinking, has upended clinical nutrition practice. Based on this evidence, recent guidelines have begun to advocate progressive nutritional treatment rather than reaching nutritional goals as early as possible. However, no large RCT has shown that progressive nutrition is superior to long-term low-dose feeding.

For patients with acute respiratory distress syndrome (ARDS), lung protective ventilation strategies [low tidal volume, low plateau pressure, and appropriate positive end-expiratory pressure (PEEP)] both promote alveolar reexpansion to maintain oxygenation and prevent ventilator-induced lung injury. It has been proven to reduce mortality and ventilator days in ARDS patients (16). The gastrointestinal (GI) tract is similar to the lungs in that it seems disadvantageous to abandon or overuse, and protective use seems to be an optimal state, which not only plays the function of the organ but also avoids the damage caused by overuse. We will combine the latest research results, summarize the idea of protective nutrition, and discuss from the three aspects of why, what and how to protect.

## 2 Why to protect

### 2.1 Full nutrition is deleterious

The estimation of caloric requirements in critically ill patients typically relies on energy expenditure (EE). However, during the acute phase of critical illness, caloric needs may be lower than EE. This discrepancy arises because the body generates endogenous glucose in the state of critical illness, a process that remains unaffected by exogenous supplementation (17). Given the endogenous production of glucose, full nutrition during the acute phase can lead to overfeeding, potentially exerting negative impacts on both the body and disease recovery (18, 19).

As early as 2011, the idea of early full nutrition was challenged. The EPaNIC trial (*N* = 4,640) compared the outcomes of critically ill patients receiving early (<48 h) supplemental parenteral nutrition (SPN) vs. late (>48 h) SPN in the ICU (9). The results indicated that patients in the late SPN group had lower infection rates, fewer complications, shorter durations of mechanical ventilation, and better prognosis. Subsequent *post-hoc* analysis of the EPaNIC trial suggested that the better outcomes in the late SPN group were due to higher nutritional intake during the acute phase in the early SPN group compared to the late SPN group, and that higher early protein intake appeared a key factor influencing the observed outcomes (20). In this study, the primary factor influencing the outcomes of critically ill patients was full feeding. The adverse effects of PN were found to be dose-dependent rather than route-dependent (20). The CALORIES trial (*N* = 2,400) also did not observe any effect of feeding route on the 30-day mortality rate of critically ill patients (21). Furthermore, the NUTRIREA-2 trial (*N* = 2,410) validated this viewpoint, finding that compared to enteral nutrition (EN), isocaloric parenteral nutrition (PN) had no differential impact on the prognosis of critically ill patients with mechanical ventilation and shock, and identified a higher incidence of digestive system complications in the EN group (13). In other clinical studies, despite variations in interventions, timing, and patient populations, the negative impact of early full nutrition on GI adverse events, blood glucose levels, and mortality has been observed (10, 11, 22). In the PROTINVENT retrospective study, it was also observed that patients receiving full nutrition (protein intake >1.2 g/kg/day) exhibited poorer clinical outcomes (23). In the NUTRIREA-3 trial (*N* = 3,044), while no increase in mortality was observed with early standard calorie and protein supplementation (calorie: 6 kcal/kg/day, protein: 0.4 g/kg/day) compared to early low-dose nutrition (calorie: 25 kcal/kg/day, protein: 1-1.3 g/kg/day) in mechanically ventilated critically ill patients with shock, there was an associated increase in ICU length of stay, gastrointestinal adverse events, and other complications (24). Higher protein supply was found in the EFFORT trial (*N* = 1,301) to be particularly harmful for patients with acute kidney injury and higher organ failure scores at baseline (25). A recent PRECISe trial (*N* = 935) found that high enteral protein supply led to poorer health-related quality of life in critically ill patients and did not improve functional outcomes within 180 days of ICU admission (26). Interestingly, the EFFORT Trial (*N* = 2,088) in non-critically ill found that compared with the control group (mean calorie 1,211 kcal/day, protein 47 g/day), A higher nutrient supply (mean calorie 1,501 kcal/day, protein 57 g/day) improved clinical outcomes in patients at nutritional risk. Another RCT found that adult patients undergoing cardiac surgery with cardiopulmonary bypass who received 2.0 g/kg/day of amino acids did not experience an increased 30-day mortality compared to the placebo group. Moreover, it reduced the incidence of acute kidney injury (AKI) (27). These outcomes appeared contradictory to previous theories. However, it should be noted that the study population did not consist of critically ill patients and is weakly comparable to previously presented studies. This also suggests that strengthening nutritional support may be beneficial for patients entering the recovery phase of critical illness. Further research is warranted to elucidate nutritional therapy in critically ill patients and to provide more comprehensive evidence for nutritional practice.

### 2.2 Moderate nutrition may be beneficial

Due to the stress state, increased metabolic rate, and the demands of tissue repair, critically ill patients have a greater need for nutritional therapy compared to ordinary patients. The supplementation of exogenous energy and protein is likely important at some point. Failure to provide adequate and timely nutrition could lead to malnutrition and other adverse clinical outcomes (28–30). In retrospective studies, an intake below 50% was found to correlate with poorer clinical outcomes. This level of intake may result in significant calorie deficit, deplete energy reserves, reduce lean body mass, and potentially increase the risk of infectious complications (8, 31). A prospective observational study across 21 countries involving 167 intensive care units (*N* = 2,772) indicated that lower energy and protein delivery were associated with increased 60-day mortality and prolonged duration of mechanical ventilation in patients with mechanical ventilation (6). However, these non-interventional studies appear to overlook the true underlying reasons for the higher mortality rate in patients with low nutritional intake [greater disease severity potentially leading to increased occurrence of feeding intolerance (FI)]. Zusman et al.'s study found (*N* = 1,171) that 60-day mortality was lowest in severely ill patients when the percent of administered calories divided by resting energy expenditure was equal to 70% (32). The EuroPN study (*N* = 1,172) demonstrated that compared to lower intake levels (<10 kcal/kg, <0.8 g/kg), a daily moderate intake (10–20 kcal/kg, 0.8–1.2 g/kg) was associated with a higher likelihood of successful weaning and a reduced risk of mortality (33). Similar to the results of a meta-analysis (34), the results of this study also support the existence of a “U-shaped curve” of nutritional intake (both excessive and inadequate supply being detrimental, with moderate intake being optimal), highlighting that moderate nutritional intake is beneficial for critically ill patients. However, as critical illness recovery progresses, the inflection point of the U-shaped curve may shift rightward (indicating higher nutritional intake). The PROTINVENT study (*N* = 455) suggested a time-dependent relationship between protein intake and mortality, revealing that the lowest 6-month mortality rates were observed when protein intake increased from <0.8 g/kg/day on days 1–2 to 0.8–1.2 g/kg/day on days 3–5, and exceeded 1.2 g/kg/day after day 5 (23).

Unfortunately, there are currently no RCTs specifically comparing the effects of low-dose and moderate-dose nutrition on the prognosis of critically ill patients. However, we believe that the most appropriate feeding dose for patients may also have a time-dependent nature, with a transition from limited to progressive to open feeding strategy, which may be more suitable for critically ill patients (35). The timing of implementing this transition in feeding strategies remains unclear, and further research is needed to identify biomarkers indicating the critical illness phase. Compared with calorie, protein lacks a two-stage or multi-stage nutritional target, which may be more conducive to precision nutritional treatment for critically ill patients.

## 3 What to protect

### 3.1 Gastrointestinal protection

Early EN (<48 h) exerts a protective effect on the GI mucosal barrier, promotes gastrointestinal peristalsis, reduces bacterial translocation, and stimulates increased intestinal blood flow, thereby aiding in the maintenance of normal metabolic activity and repair capacity of the intestinal mucosa (36, 37). Under the blow of critical illness, protective nutrition may be a good match for impaired GI function, which not only plays the role of GI tract, but also avoids the deterioration of GI function. The ESPEN guidelines recommend that the feeding volume of early EN in the acute phase of critical illness should not exceed 70% of estimated EE (1, 19), and a progressive feeding approach appears to be more beneficial for the clinical outcomes of critically ill patients (18, 35).

Animal experiments indicate that low-dose EN promotes the recovery of intestinal barrier function in rats following ischemia/reperfusion injury by enhancing NF-κB/HIF-1α pathway expression (38). Furthermore, low-dose EN can also activates the JAK1-STAT6 pathway, facilitating the expression of pIgR and secretory immunoglobulin A (sIgA), thereby mitigating immune damage to the murine intestinal mucosa (39). Robles et al. summarized overfeeding in animal experiments. Overfeeding can lead to a strong and prolonged hyperphagic response, and it may reduce survival following infection (40). In addition, Zhang et al. found in a mouse model of acute pancreatitis that Short-peptide-based EN has the function of restoring ZO-1 expression, mucous layer and goblet cells, thereby reducing intestinal bacterial translocation in mice with severe acute pancreatitis (41).

Unfortunately, there is currently no large-scale RCT that has found early EN to be truly superior to delayed EN (42). There is no evidence to suggest that early EN reduces mortality in critically ill patients. The differences observed in secondary outcomes such as ICU length of stay and infections vary greatly across studies, and there remains significant heterogeneity (42). In fact, early EN may not be suitable for all critically ill patients, as initiating it too early may lead to FI. Predicting the likelihood of FI and implementing early pre-intervention (delayed EN) for high-risk patients is a more conservative strategy that may reduce the incidence of FI (43). However, delayed EN may fails to provide the trophic effects on the gut. It is clear that early EN does not have a universally positive effect on all patients; thus, an individualized assessment of the benefits and risks of early EN is warranted. Accordingly, further research in this field is warranted.

### 3.2 Nutritional protection

During the acute phase of critical illness, patients often experience not only impaired gastrointestinal motility and barrier function but also digestive and absorptive dysfunctions (1). A prospective cross-sectional study (*N* = 563) revealed that, excluding primary exocrine pancreatic insufficiency (EPI), 52.2% of critically ill patients exhibited EPI (fecal elastase −1 < 200 ug/g), with 18.3% of patients experiencing severe EPI (fecal elastase −1 < 100 ug/g). Factors such as shock, sepsis, diabetes, cardiac arrest, hyperlactatemia, invasive mechanical ventilation, and hemodialysis may all contribute to the development of EPI, severely impacting the digestive absorption capacity of critically ill patients (44). The deficiency of digestive enzymes may leads to the restriction of protein digestion in severe patients, and the protein supplemented through the EN pathway is not easily absorbed by the intestine. Therefore, there seems to be an irreconcilable contradiction between protein requirements and digestion and absorption disorders. Inappropriate nutritional support may lead to adverse gastrointestinal events such as abdominal distension and diarrhea (45).

In 2023, the Mayo Clinic defined a short peptide-based formula (PBF) as an EN formula in which protein is hydrolyzed into “2-3 peptides.” 2-3 peptides can enter intestinal epithelial cells through active transport and are the main way the body absorbs proteins. PepT1 operates through a proton-coupled mechanism, utilizing the proton gradient to drive the uptake of 2-3 peptides without the need for additional energy expenditure (46). When a proton and a peptide simultaneously bind to the external surface of PepT1, a conformational change occurs, resulting in the translocation of the bound peptide and proton into the cell. This mechanism allows PepT1 to function efficiently in the low pH environment of the intestinal lumen (46–48). Another distinction between PBF and standard polymerized formula (SPF) is that the lipid component of SPF consists predominantly of long-chain triglycerides (LCT), typically ranging from 13 to 24 carbon atoms. These LCTs necessitate pancreatic and biliary fat digestion and emulsification to be metabolized into glycerol and free fatty acids, ultimately entering enterocytes and subsequently forming chylomicrons (49). In contrast, PBF often contains significant amounts of medium-chain triglycerides (MCT), with lengths of 6–12 carbon atoms. MCTs are passively absorbed, bypassing the need for complex fat digestion and emulsification (50). It has also been shown the MCTs do not rely on the carnitine acyltransferase system to enter mitochondria for β-oxidation (51). This enables quicker metabolism of MCTs and enhances their utilization, even in conditions of protein deficiency (52). Obviously, these may be beneficial for critically ill patients. Therefore, this formula was suggested to improve EN tolerance, and it was found that the use of PBF in patients with FI may lead to improved clinical outcomes, along with a corresponding reduction in healthcare utilization and potential nursing costs (53). Due to the ileal brake mechanism, critically ill patients receiving jejunal feeding are unable to stimulate the secretion of pancreatic proteases (54). Therefore, compared to other formulas, PBF may be more suitable for patients receiving post-pyloric feeding (55). Additionally, due to mitochondrial dysfunction and tissue hypoxia in the acute phase of critical illness, lipid metabolism is impaired (56). Elevated levels of exogenous fat nutrition can exacerbate mitochondrial damage and organ dysfunction, potentially leading to poorer clinical outcomes (57–59). Early provision of low-fat nutrition may play a protective role in nutritional support.

In some clinical studies, PBF has been found to reduce the incidence of gastric retention and diarrhea, achieve nutritional adequacy more rapidly, shorten ICU length of stay, and lower readmission rates (60–64). The ASPEN guidelines recommend the use of PBF for critically ill patients with severe malabsorption, such as persistent diarrhea (4). However, due to the low quality of evidence supporting these recommendations, they are considered as having a low level of evidence. Nevertheless, based on current theoretical rationale, there is reason to believe that PBF can provide this nutritional protective effect for patients with gastrointestinal injury, although high-quality research is still needed to further validate its efficacy.

### 3.3 Metabolic protection

#### 3.3.1 Metabolism in the acute phase of critical illness

During the acute phase of critical illness, particularly in the early stages, the body predominantly undergoes catabolism (1). This phase is characterized by accelerated protein and fat breakdown, increased gluconeogenesis, insulin resistance, and elevated basal and resting EE. Due to the body's self-protection mechanisms, in response to critical illness, a significant amount of protein and fat is converted into glucose to meet the metabolic demands (65). More than half of the body's energy needs are supplied through this pathway, which remains active regardless of exogenous energy supplementation (17, 19, 66). Consequently, there exists a risk of overfeeding during this phase, potentially inhibiting certain beneficial metabolic processes.

#### 3.3.2 Mitochondrial protection

During the acute phase of critical illness, varying degrees of mitochondrial dysfunction are often present (67). Mitochondria serve as the primary site for cellular energy production, and impairment of their function can lead to disturbances in cellular metabolism and organ dysfunction (68). Mitochondria are particularly sensitive to the increased oxidative stress in critical illness. Abnormalities in the function and structure of this organelle further lead to excessive generation of reactive oxygen species and decreased production of adenosine triphosphate (ATP) (69, 70). To reduce metabolic demands, mitochondrial dysfunction may progress in a manner similar to hibernation, which could aid in maintaining cellular life, though it may come at the cost of organ system failure (67). In such circumstances, the administration of full nutrients may have deleterious effects, as damaged mitochondria are unable to effectively utilize the additional energy supply, potentially leading to increased oxidative stress and cellular damage (71–73). Therefore, in clinical practice, protective nutrition may be more appropriate for severe patients during the acute phase to avoid exacerbating mitochondrial dysfunction and the associated adverse outcomes. It is not only a strategy of nutritional protection but also a strategy of organelle and organ protection. The objective of this strategy is to balance energy provision with the metabolic needs of patients by controlling nutrient intake, while simultaneously reducing the risk of exacerbating pathological processes resulting from metabolic disturbances and oxidative stress.

#### 3.3.3 Autophagy activation

Autophagy, as a central molecular pathway, plays a crucial role in maintaining cellular and organismal homeostasis (74, 75). Through this process, cells are able to eliminate damaged or dysfunctional organelles and proteins, thereby preserving the stability of the cellular environment. In critically ill states, cells often suffer varying degrees of functional impairment, which can increase the risk of multiple organ failure. Therefore, the activation of autophagy becomes particularly important (76). Animal experiments have demonstrated the protective effects of autophagy on kidney, lung, liver, and intestinal injuries (77–80). However, artificial feeding can inhibit the activation of autophagy in critical illness, particularly at high protein/amino acid doses (18). Insufficient autophagy may further exacerbate mitochondrial dysfunction, leading to organ failure and adverse outcomes (81, 82). During the acute phase of critical illness, protective nutrition establishes a relatively starved or nutrient-restricted condition, thereby promoting the beneficial metabolic process of autophagy. This approach may potentially improve the prognosis of critically ill patients.

#### 3.3.4 Ketogenic activation

Under fasting or starvation conditions, due to inadequate glucose supply, the body undergoes a gradual transition from glucose metabolism to lipid metabolism, accompanied by the activation of ketogenesis (83, 84). Ketogenesis primarily functions through the inhibition of histone deacetylases, reduction of oxidative stress, enhancement of mitochondrial efficiency, promotion of autophagy, and modulation of inflammation (83, 85–87). Studies have shown that ketogenesis can increase the oxidation of intramuscular triacylglycerol during exercise, thereby increasing endurance (88). Animal experiments indicate that deficiency in peroxisome proliferator-activated receptor alpha (PPARα) impairs ketogenesis activation, which is associated with increased mortality in mice following bacterial infection (89). Studies have shown that ketones can provide up to 70% of the brain's energy needs, that the uptake of ketones by the brain increases significantly during acute brain injury, and that ketone supplements have potential in the treatment of traumatic brain injury (90). Therefore, for patients at high risk of FI, opting for early fasting with late initiation of small-dose EN appears to offer greater benefits. We should probably not focus solely on the balance between the benefits of early EN and the harms of FI.

#### 3.3.5 Blood glucose control

Factors such as nutritional therapy and stress during critical illness can lead to elevated blood glucose, but the optimal blood glucose target is still controversial (91). Several previous studies have shown that early administration of PN with tight glycemic control (TGC) between 80 and 110 mg per deciliter reduces morbidity, ICU length of stay, and mortality in surgical ICU patients (92, 93). However, in a recent RCT by Gunst et al. (*N* = 9,230), TGC did not reduce mortality among critically ill patients who did not receive early PN (94). Only minor benefits of TGC were observed in several secondary outcomes. The seemingly contradictory findings stemmed from the fact that delayed initiation of PN implied lower energy intake, thereby resulting in significantly less severe hyperglycemia in the liberal glucose control group compared to previous two RCTs (94). We have reason to believe that the higher mortality observed in the control groups of the previous two RCTs may have been due to overfeeding during the acute phase and more severe hyperglycemia. This could lead to glucose overload in critical organs and cells, which is associated with poorer clinical outcomes (95). Studies have shown that higher blood glucose may inhibit mitochondrial repair processes, leading to further mitochondrial dysfunction (81).

During critical illness, stress and emergency responses can increase the activity of the neuroendocrine system, promoting the release of catecholamines and cortisol. This stimulation enhances glycogenolysis and gluconeogenesis in the liver, concurrently inducing peripheral insulin resistance (IR) and inhibiting glucose entry into cells (96). Additionally, inflammatory responses during critical illness contribute to IR (97). IR is characterized by reduced effectiveness of insulin in facilitating glucose uptake and utilization, along with downregulation of insulin-dependent glucose transporter proteins in peripheral tissues, thereby leading to stress-induced hyperglycemia (98). The triglyceride-glucose (TyG) index, calculated from fasting triglyceride (TG) and fasting plasma glucose (FPG) levels, has been validated for assessing the degree of IR in patients (99–101). Moreover, the excessive use of insulin due to hyperglycemia can also result in poorer clinical outcomes (102). In one RCT (*N* = 6,104), the TGC group exhibited higher mortality compared to the control group, attributable to the increased administration of insulin (103). Additionally, insulin may adversely affect organ recovery and disease resolution by inhibiting autophagy and ketogenesis (104, 105). Protective nutrition can not only reduce the incidence of hyperglycemia, but also reduce the use of insulin, which may be more suitable for severe patients.

#### 3.3.6 The interaction of metabolic processes

**Figure 1:**
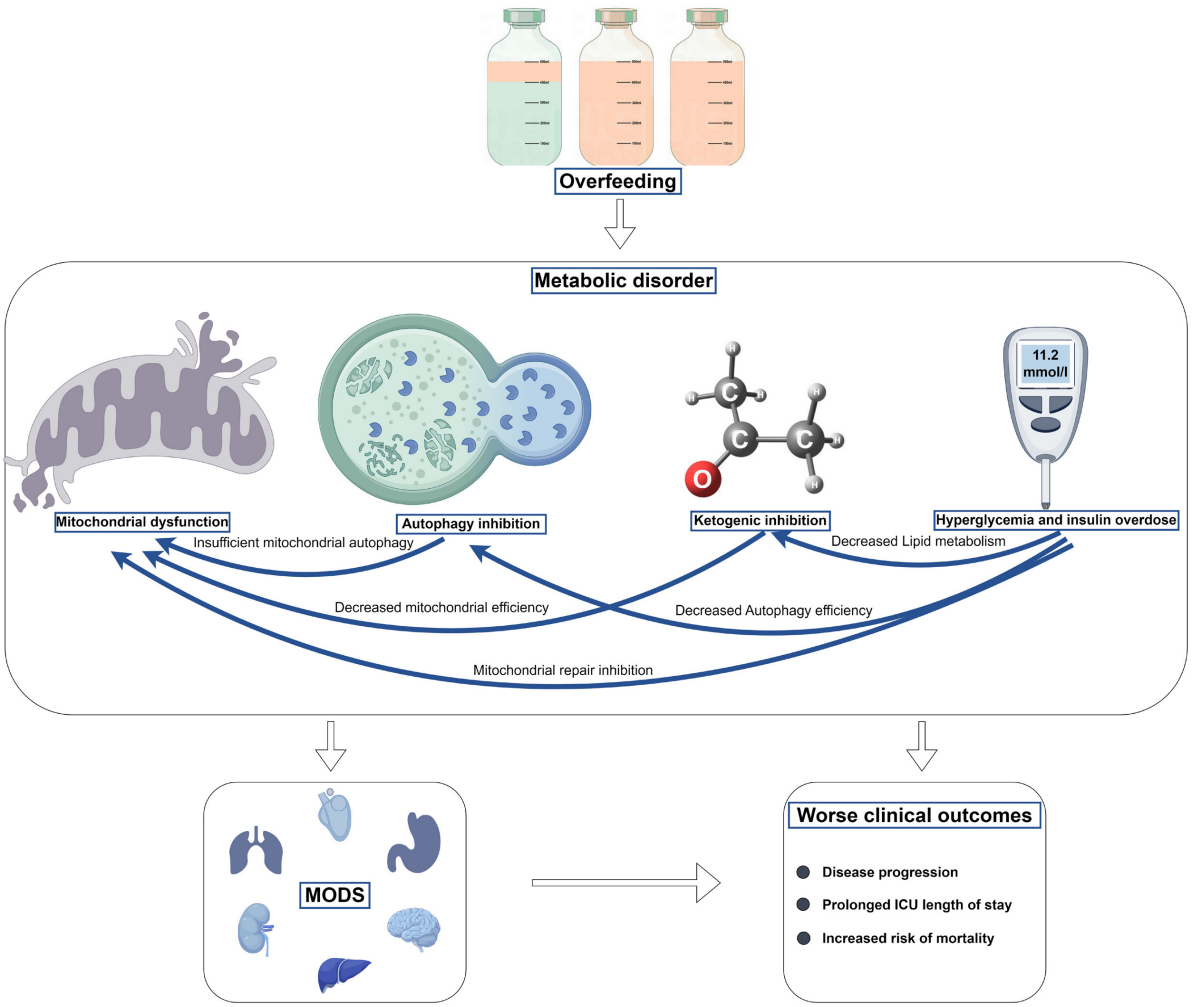
The harm of overfeeding in the acute stage of critical illness.

Overfeeding can lead to adverse effects such as mitochondrial dysfunction, reduced autophagy, ketogenesis suppression, hyperglycemia, and excessive insulin utilization. As outlined previously, these metabolic processes interact intricately. For instance, during overfeeding, elevated blood glucose levels can inhibit mitochondrial repair, thereby exacerbating mitochondrial dysfunction. Moreover, it can decrease autophagic efficiency, leading to reduced autophagy, and impair fatty acid metabolism, thereby suppressing ketogenesis. These interrelationships among metabolic processes may exacerbate organ damage and result in poorer outcomes (Figure 1). Theoretically, protective nutrition strategies are more beneficial in activating these beneficial metabolic processes to benefit critically ill patients.

## 4 How to protect

How can we practice to achieve the GI protection, nutritional protection and metabolic protection that we mentioned earlier? Based on the above theoretical basis, and compared with the lung protective ventilation strategy, we put forward the protective nutrition strategy.

**Figure 2:**
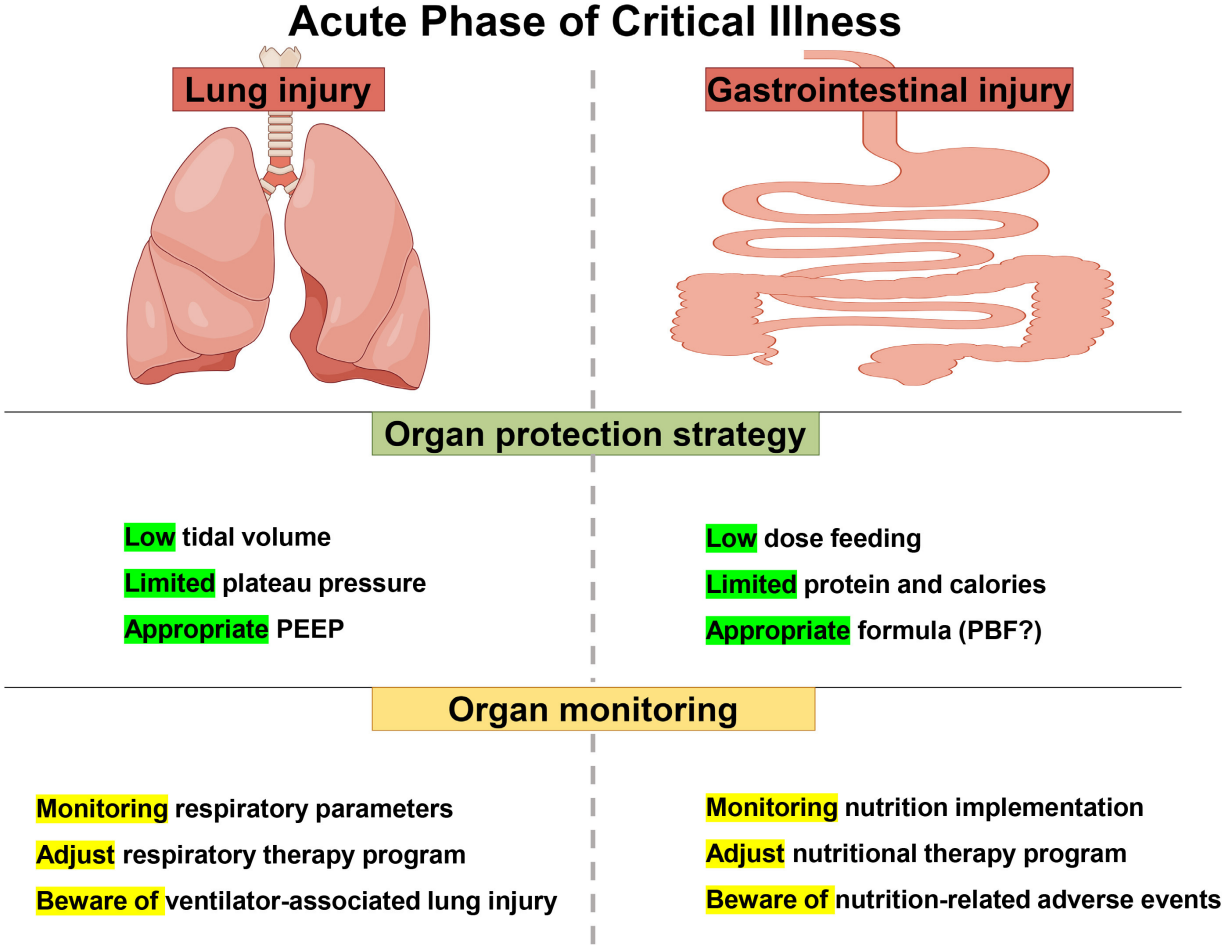
Lung protective ventilation strategies and protective nutrition strategies in acute stage of critical illness.

Low-dose feeding and low-dose protein and calories strategies are similar, utilizing limited GI function while avoiding the harms of overfeeding. These two strategies are similar to low tidal volume and limited platform pressure in lung protective ventilation strategies for ARDS patients, which take advantage of limited lung function and avoid the harm caused by excessive ventilation. On the other hand, appropriate formula may reduce the burden of GI digestion and absorption, lower the risk of a secondary blow to GI, and may decrease the occurrence of FI, although the current evidence on the selection of nutritional formulas is very weak. This strategy would be similar to appropriate PEEP, which promotes collapsed alveolar distension and improves ventilate blood flow ratio and oxygenation. It can also reduce lung damage caused by overinflated alveoli (Figure 2).

Current guidelines still recommend early EN for patients who are able to tolerate it, in order to achieve the benefits of EN (19). When EN alone is insufficient to meet the patient's nutritional needs, SPN can be used alongside EN to fulfill the patient's nutritional needs, although the optimal timing for SPN application remains unclear (4, 19). When a patient is unable to tolerate EN, the benefits and risks of PN should be carefully weighed, and PN may need to be provided in a timely manner, rather than abandoning nutritional therapy. The potential harm from overfeeding may be more severe than the risks associated with the nutritional route itself. It is important to focus on the daily nutrient intake and the monitoring of overfeeding.

We have to admit that it is difficult to come up with a universal strategy due to the large individual differences in critically ill patients. Although we can only come up with a general idea of nutritional treatment, following these recommendations may benefit patients.

## 5 What needs to be monitored

### 5.1 Overfeeding

The monitoring of overfeeding is a hot topic in recent years, because we recognize the harm of overfeeding and try to avoid this phenomenon in clinical nutrition practice (106). Indirect calorimetry serves as the gold standard for measuring EE, derived from measurements of VO2 and VCO2 (107). However, the measured EE does not necessarily reflect the true energy requirements of patients in the acute phase of critical illness, during which significant endogenous glucose production occurs (17). Providing nutrition equivalent to measured EE often results in overfeeding (1, 36). Currently, the clinical measurement of endogenous glucose production is complex and not widely implemented. Achieving the equation of endogenous glucose + exogenous supplementatio*n* = EE is not our ultimate goal, as optimal exogenous supplementation may also need to consider the potential risks of inhibiting fasting responses, such as autophagy and ketosis. While the ideal dose is individualized and still uncertain, identifying clinical indicators of overfeeding is crucial for guiding nutritional therapy effectively (18).

Overfeeding can manifest as hyperglycemia and increased insulin requirements, azotemia, elevated urea-to-creatinine ratio (UCR), and hypertriglyceridemia. However, these indicators are non-specific and can vary between individuals (106). Interestingly, underfeeding can also manifest as hyperuremia and an elevated blood UCR (108). UCR is also influenced by factors such as circulating fluid volume, underlying disease and therapeutic intervention, which limits its specificity as a reliable indicator of overfeeding (109, 110). This presents significant challenges in using UCR as an effective marker for overfeeding. In critically ill patients, there is an urgent need to explore and develop correction equations for UCR that accurately reflect nutritional status. The correlation between UCR and nutritional intake remains an area requiring further research. Currently, significant challenges exist in monitoring overfeeding due to the lack of specific indicators. We advocate for the concept of protective nutrition, as the harm from overfeeding may surpass the detriments of underfeeding (111). In the absence of adequate monitoring tools, the administration of small doses of nutrition during the early acute phase is recommended, at least not to cause more harm.

### 5.2 Refeeding syndrome

Refeeding syndrome (RS) refers to a spectrum of metabolic abnormalities that occur when nutritional intake is reintroduced after a period of prolonged starvation or malnutrition. It is primarily characterized by metabolic changes, electrolyte imbalances, and vitamin deficiencies following the initiation of nutritional therapy (112). Micronutrients can distribute unevenly during critical illness, rendering early measurements potentially misleading (113). In the context of refeeding syndrome, which occurs upon the rapid reinstitution of feeding after prolonged starvation, declines in micronutrient and electrolyte concentrations (e.g., vitamin B1, phosphate, potassium) can be abrupt and severe, potentially posing a fatal risk for individuals in a state of starvation or catabolic metabolism (114, 115). Micronutrients play crucial roles in metabolism, immunity, gene transcription, and other physiological processes (116). However, symptoms of micronutrient deficiencies often mimic those of critical illness, leading to frequent oversight (117). In the context of refeeding syndrome (RS, which can occur after the rapid reinstitution of feeding following prolonged starvation), micronutrient deficiencies may manifest. The current common diagnostic criterion for refeeding syndrome includes a reduction in phosphate levels to < 0.65 mmol/L, with a decrease of at least 0.16 mmol/L (118). An RCT (*N* = 339) by Doig et al. showed that adult critically ill patients who developed refeeding hypophosphatemia within 72 h of starting nutritional support in the ICU had better 60-day survival and longer overall survival in the restricted feeding group than in the standard feeding group (118). A retrospective study by Olthof et al. (*N* = 337) showed that low caloric intake was associated with a reduced risk of death at 6 months in patients with refeeding hypophosphatemia (119). Therefore, monitoring phosphate changes, early identification of refeeding hypophosphatemia, and giving targeted restrictive nutrition and correcting micronutrient and electrolyte deficiencies may improve the prognosis of severely ill patients (112). Additionally, the timely supplementation of micronutrients is crucial. The 2022 ESPEN guidelines propose recommendations for the presumed optimal intake of micronutrients (113). However, the evidence supporting these recommendations is relatively weak. Due to the uneven distribution of micronutrients caused by factors such as disease and inflammation, relying on blood micronutrient concentrations as a basis for supplementation is not accurate. Furthermore, there is no evidence of the benefits of excessive supplementation, and there may be harm (113, 120). The ideal dosage for micronutrient supplementation remains an area requiring further investigation.

### 5.3 Acute mesenteric ischemia

Acute mesenteric ischemia (AMI) is infrequently encountered in the ICU but carries a markedly high mortality rate (exceeding 50%) (121). Despite recent advancements in the recognition of AMI, involving imaging and interventional radiology for diagnostic assistance, there has been limited improvement in mortality (121, 122). Delayed diagnosis leading to delayed treatment likely contributes significantly to this scenario, representing a critical prognostic factor (123, 124). Early identification of AMI, prompt cessation of EN, implementation of intestinal revascularization or surgical intervention can prevent further exacerbation of intestinal ischemia, thus avoiding or delaying the occurrence of intestinal necrosis (125, 126). AMI should be ruled out when patients present with acute gastrointestinal symptoms that cannot be explained by feeding alone. A meta-analysis summarizing biomarkers for diagnosing AMI found that urinary I-FABP and D-dimer exhibit moderate predictive values in assessing transmural mesenteric ischemia. Unfortunately, none of the biomarkers reached the level of accurate prediction (122). Whether combining these biomarkers will increase diagnostic effectiveness still needs to be explored further. When there is a high suspicion of AMI, computed tomography imaging, particularly a biphasic protocol consisting of angiography and venous phase scanning, is widely utilized to confirm nonspecific clinical findings (127). Raising awareness about AMI and its early identification and diagnosis is crucial for improving the prognosis of critically ill patients.

### 5.4 Other gastrointestinal adverse events

Monitoring during nutritional implementation is crucial because it is a personalized therapy, and all plans are not static. We should emphasize the monitoring of the nutritional implementation process to achieve comprehensive protective nutrition. Monitoring of nutrition is similar to monitoring of respiratory therapy, and only continuous monitoring and continuous adjustment could achieve continuous protection (Figure 2).

Monitoring of GI adverse events is essential, and factors such as bowel sounds, intra-abdominal pressure, and bedside ultrasound can provide valuable indications of the patient's GI function status. Due to limited monitoring methods, our initial assessment of the GI function of critically ill patients may be erroneous, which is unavoidable. Fortunately, monitoring can play a role in timely correction, minimizing the harm caused by erroneous decisions.

## 6 Conclusions

Recent guidelines and RCTs have shown that overfeeding in the acute phase is detrimental to clinical outcomes in severely ill patients. Protective nutrition strategies are a synthesis of guidelines and clinical studies. We also discussed the reasons for implementing protective nutrition strategies, and discussed its potential beneficial effects on GI function, metabolic complications, and organ function. In addition, we highlight the importance of post-nutritional monitoring, which, despite its relative scarcity, is necessary to implement protective nutrition throughout. There is significant individual variability among critically ill patients, and a universal feeding strategy applicable to all patients is difficult to obtain, but the concept of protective nutrition is what we advocate.
